# Allylpropyldisulfid: MAK-Begründung, Nachtrag

**DOI:** 10.34865/mb217959d10_1ad

**Published:** 2025-03-31

**Authors:** Andrea Hartwig, MAK Commission

**Affiliations:** 1 Institut für Angewandte Biowissenschaften. Abteilung Lebensmittelchemie und Toxikologie. Karlsruher Institut für Technologie (KIT) Adenauerring 20a, Geb. 50.41 76131 Karlsruhe Deutschland; 2 Ständige Senatskommission zur Prüfung gesundheitsschädlicher Arbeitsstoffe. Deutsche Forschungsgemeinschaft, Kennedyallee 40, 53175 Bonn, Deutschland. Weitere Informationen: Ständige Senatskommission zur Prüfung gesundheitsschädlicher Arbeitsstoffe | DFG

**Keywords:** Allylpropyldisulfid, Reizwirkung

## Abstract

The German Senate Commission for the Investigation of Health Hazards of Chemical Compounds in the Work Area (MAK Commission) has re-evaluated the data for allyl propyl disulfide [2179-59-1] with regard to its occupational exposure limit value (maximum concentration at the workplace, MAK value) and all other toxicological end points. Relevant studies were identified from a literature search. Allyl propyl disulfide causes irritation of the eyes, nose and throat in humans. There are no new data on irritation relevant for evaluation. As the previous derivation of the MAK value for allyl propyl disulfide does not correspond to the current procedure of the Commission, the MAK value and the peak limitation are suspended and the substance is listed in the Section II b of the List of MAK and BAT Values. Allyl propyl disulfide is not mutagenic in Salmonella typhimurium and there are no indications of an independent sensitizing effect. Skin contact is not expected to contribute significantly to systemic toxicity. Data on developmental toxicity, carcinogenicity and germ cell mutagenicity are not available.

**Table:** 

**MAK-Wert**	**nicht festgelegt, vgl. Abschnitt II b der MAK- und BAT-Werte-Liste**
**Spitzenbegrenzung**	**–**
	
**Hautresorption**	**–**
**Sensibilisierende Wirkung**	**–**
**Krebserzeugende Wirkung**	**–**
**Fruchtschädigende Wirkung**	**–**
**Keimzellmutagene Wirkung **	**–**
	
BAT-Wert	–
	
Synonyma	2-Propenylpropyldisulfid Propylallyldisulfid
Chemische Bezeichnung (IUPAC-Name)	1-(Prop-2-enyldisulfanyl)propan
CAS-Nr.	2179-59-1
Formel	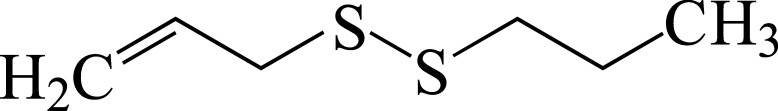
	C_6_H_12_S_2_
Molmasse	148,12 g/mol
Schmelzpunkt	verfestigt sich bei –15 °C (NIOSH 1992)
Siedepunkt bei 17,3 hPa	78−80 °C (NIOSH 1992)
Dichte bei 15 °C	0,93 g/cm^3^ (NIOSH 1992)
Dampfdruck bei 25 °C	0,52 hPa (ber.; NCBI 2024)
log K_OW_	2,4 (ber.; NCBI 2024)
Löslichkeit	unlöslich in Wasser, löslich in Ether, Kohlenstoffdisulfid und Chloroform (NIOSH 1992)
**1 ml/m^3^ (ppm) ≙ 6,164 mg/m^3^**	**1 mg/m^3^ ≙ 0,163 ml/m^3^ (ppm)**
	
Hydrolysestabilität	k. A.
Verwendung	Allylpropyldisulfid ist ein in Nahrungsmitteln natürlich vorkommender Stoff (Hauptkomponente der ätherischen Öle von Zwiebel und Knoblauch) (Henschler 1979) und ein Lebensmittelzusatzstoff (WHO 2008).

Es liegen eine Begründung (Henschler 1979) und ein Nachtrag (Greim 2002) zur Spitzenbegrenzung vor. In diesem Nachtrag werden die Ableitung des MAK-Wertes überprüft und alle weiteren toxikologischen Endpunkte bewertet.

Allylpropyldisulfid ist im Rahmen von REACH vorregistriert, es liegen jedoch keine Registrierungsdaten vor (ECHA 2023). Die Verwendung als Lebensmittelzusatzstoff ist unbedenklich (WHO 2008).

## Allgemeiner Wirkungscharakter

Allylpropyldisulfid führt beim Menschen zu Reizungen von Augen, Nase und Kehle.

An Salmonella typhimurium wirkt Allylpropyldisulfid nicht mutagen.

Hinweise auf eine eigenständige sensibilisierende Wirkung von Allylpropyldisulfid liegen nicht vor.

## Erfahrungen beim Menschen

Bei Konzentrationen von 1,7–3,4 ml/m^3^ traten bei Arbeitern Reizwirkungen auf **(**Henschler 1979).

Außer zur sensibilisierenden Wirkung gibt es zu keinem Endpunkt neue Daten.

### Hautsensibilisierende Wirkung

**Abb.1:**
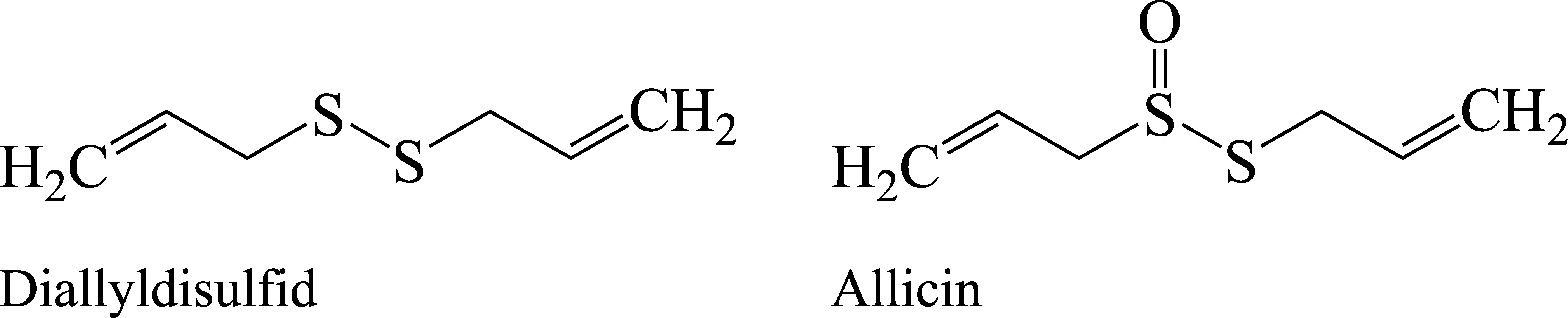
Strukturformeln von Diallyldisulfid und Allicin

Beruflich bedingte Kontaktekzeme der Hände, aber auch aerogene Kontaktekzeme durch Knoblauchpulver wurden beschrieben, wobei Allylpropyldisulfid neben Diallyldisulfid und Allicin (siehe Abbildung 1) ein Hauptallergen in Knoblauch darstellt (Bauer et al. 2018; Papageorgiou et al. 1983). Die Allergene scheinen in geringerer Konzentration auch in Zwiebeln vorhanden zu sein (Bahna 2004; Van Hecke 1977).

Informationen über die in Knoblauch und Zwiebeln enthaltenen Mengen der einzelnen Allergene liegen nicht vor. 

In einer Studie wurde die allergene Wirkung von Allylpropyldisulfid untersucht. An 23 Freiwilligen, die gegen Knoblauch sensibilisiert waren, wurden verschiedene Sulfide und Disulfide epikutan getestet. Hierbei ergaben sich mit einer 0,5%igen Testzubereitung von Allylpropyldisulfid in Vaseline am 2. Tag nach der Applikation positive Reaktionen bei sechs von 23 Personen, mit einer 5%igen Testzubereitung reagierten alle 23 Personen positiv (Papageorgiou et al. 1983). Es liegen keine Angaben zur Reaktionsstärke vor. 

Weiterhin wurden sieben Freiwillige mit verschiedenen aufkonzentrierten Fraktionen der säulenchromatographischen Trennung eines ethanolischen Knoblauch-Extraktes getestet. Die Ablesung erfolgte am 2. Tag. Die Fraktionen, die positive Reaktionen auslösten, enthielten Diallyldisulfid (Papageorgiou et al. 1983). Angaben zu Allylpropyldisulfid fehlen. 

Es liegen eine Reihe weiterer Berichte über durch Knoblauch oder Zwiebeln bedingte allergische Kontaktekzeme der Hände, selten auch aerogene Kontaktekzeme sowie Symptomatiken einer Typ-I-Allergie vor, wobei in diesen Untersuchungen keine Aufschlüsselung in allergieauslösende Stoffe vorgenommen wurde. Gelegentlich wurden in diesem Zusammenhang Epikutantests mit Diallyldisulfid durchgeführt (z. B. Hubbard und Goldsmith 2005; Kanerva et al. 1996; Vester et al. 2012; Einzelfälle z. B. Moyle et al. 2004; Pérez-Calderón et al. 2002; Sinha et al. 1977).

Zusammengefasst weisen die vorliegenden Daten auf eine allergene Wirkung von Knoblauch und Zwiebeln hin, jedoch lässt sich aus diesen Daten aufgrund der Mischexposition nicht auf ein allergenes Potenzial von Allylpropyldisulfid schließen.

### Atemwegssensibilisierende Wirkung

Zur atemwegssensibilisierenden Wirkung von Allylpropyldisulfid liegen keine Daten vor. In mehreren Publikationen wird über Typ-I-Reaktionen im Zusammenhang mit Knoblauch berichtet (z. B. Armentia et al. 2020). In diesen Studien finden sich jedoch keine Hinweise auf Allylpropyldisulfid als mögliches auslösendes Allergen. Vermutet wird die Alliinlyase als mögliches Atemwegsallergen in Knoblauch (Kao et al. 2004; van der Walt et al. 2010). Insgesamt lassen diese Studien keinen Rückschluss auf die atemwegssensibilisierende Wirkung von Allylpropyldisulfid zu. 

## Tierexperimentelle Befunde und In-vitro-Untersuchungen

Außer zur sensibilisierenden und genotoxischen Wirkung liegen keine neuen Daten vor.

### Allergene Wirkung

#### Hautsensibilisierende Wirkung

Es wurden drei tierexperimentelle Untersuchungen am Meerschweinchen durchgeführt (Tabelle 1). Zehn Hartley-Meerschweinchen wurden intradermal mit einer 10%igen Emulsion aus 0,5 g wässrigem Knoblauch-Extrakt in Wasser und Freund-Adjuvans an fünf aufeinanderfolgenden Tagen sensibilisiert. Die Provokation erfolgte 14 Tage nach der letzten Injektion als offener Epikutantest mit 25 µl des wässrigen Knoblauchextrakts. Bei der Ablesung nach 24 Stunden reagierten alle Tiere stark positiv. Das Testergebnis ist damit als positiv zu bewerten. Weitere acht Himalayan-spotted-Albino-Meerschweinchen wurden analog mit einer etwa 10%igen (G/V) Testzubereitung eines lyophilisierten Knoblauchextrakts in Ethanol intradermal sensibilisiert, die Tiere erhielten insgesamt drei Injektionen. Die Provokation erfolgte 14 Tage nach der letzten Injektion offen epikutan. Es ist unklar, wie die Tiere auf den 2%igen Ethanol-Ether-Extrakt reagierten (Widerspruch in der Publikation zwischen Text und Tabelle). Auf einen 10%igen ethanolischen Extrakt reagierten die Tiere positiv. Die Tiere wurden ebenfalls mit weiteren Testsubstanzen provoziert, dabei wurde die Dosis der Testsubstanzen so berechnet, dass sie der Molarität der 1%igen Diallyldisulfid-Testzubereitung (0,068 mol/l) entspricht. Sieben von acht Meerschweinchen reagierten positiv auf Diallyldisulfid (1%ige Testzubereitung). Auf die 1%ige Testzubereitung von Allylpropyldisulfid reagierte keines der sieben Tiere, wobei alle auf die 5%ige Testzubereitung reagierten. Eine dritte Gruppe von acht Himalayan-spotted-Albino-Meerschweinchen wurde analog mit einer etwa 1%igen (G/V) Emulsion von Diallyldisulfid sensibilisiert. Alle Tiere reagierten positiv auf die Provokation mit Diallyldisulfid (1%ige Testzubereitung), 2%igem Ethanol-Ether-Extrakt und 10%igem ethanolischen Extrakt. Auf die 1%ige Testzubereitung von Allylpropyldisulfid reagierte keines der acht Tiere, wobei alle auf die 5%ige Testzubereitung reagierten. Demnach ist eine Kreuzreaktion möglich (Papageorgiou et al. 1983).

Ein sekundär zitierter Bühler-Test mit Diallylsulfiden (Diallylmono-, -di-, -tri-, -tetrasulfid, k. w. A.) kam zu einem positiven Ergebnis. Weitere Angaben fehlen (US EPA 2003). 

**Tab. 1:** Ergebnisse der tierexperimentellen Untersuchungen am Meerschweinchen. Die Tiere wurden sensibilisiert mit der jeweiligen Testzubereitung und Freund-Adjuvans und anschließend mit verschiedenen Extrakten und Disulfiden provoziert (Papageorgiou et al. 1983)

**Provokation mit**	**Sensibilisierung mit**		
	**wässrigem Knoblauch-Extrakt**	**lyophilisiertem Knoblauchextrakt in Ethanol** **(10 %)**	**Diallyldisulfid** **(1 %)**
wässrigem Extrakt	positiv (10/10)	n. d.	n. d.
Diallyldisulfid (1 %)	n. d.	positiv (7/8)	positiv (8/8)
Ethanol-Ether-Extrakt (2 %)	n. d.	unklar^a)^	positiv (8/8)
Ethanol-Extrakt (10 %)	n. d.	positiv (7/7)	positiv (8/8)
Allylpropyldisulfid (1 %)	n. d.	negativ (0/7)	negativ (0/8)
Allylpropyldisulfid (5 %)	n. d.	positiv (7/7)	positiv (8/8)

n. d.: nicht durchgeführt

^a)^ Angaben in Tabelle und Text widersprüchlich

#### Atemwegssensibilisierende Wirkung

Hierzu liegen keine Daten vor.

### Genotoxizität

#### In vitro

Allylpropyldisulfid wirkte in drei Untersuchungen aus den 1980er Jahren in Konzentrationen bis zu 5000 µg/Platte mit und ohne Zusatz eines metabolischen Aktivierungssystems nicht mutagen an Salmonella typhimurium TA97, TA98, TA100, TA102, TA1535 und TA1537 (Eder et al. 1980, 1982; Zeiger et al. 1988). 

#### In vivo

Hierzu liegen keine Daten vor.

## Bewertung

Kritischer Effekt ist die lokale Reizwirkung beim Menschen an Augen, Nase und Kehle.

**MAK-Wert. **Der bisherige MAK-Wert für Allylpropyldisulfid von 2 ml/m^3^ ist aus den Reizerscheinungen an Schleimhäuten abgeleitet und nicht hinreichend begründet (Henschler 1979). Es liegen keine validen Langzeituntersuchungen zur Reizwirkung oder anderen Endpunkten vor, die eine Bewertung der gesundheitlichen Gefährdung zulassen. Da die bisherige Ableitung des MAK-Wertes für Allylpropyldisulfid nicht der aktuellen Vorgehensweise der Kommission entspricht, werden der MAK-Wert und die Spitzenbegrenzung aufgehoben und der Stoff dem Abschnitt II b der MAK- und BAT-Werte-Liste zugeordnet. 

**Fruchtschädigende Wirkung. **Es liegen keine Daten zur fruchtschädigenden Wirkung vor. Da kein MAK-Wert aufgestellt werden kann, entfällt die Zuordnung zu einer Schwangerschaftsgruppe.

**Krebserzeugende Wirkung. **Es liegen keine Untersuchungen und kein Strukturverdacht für eine kanzerogene Wirkung vor. Daher wird Allylpropyldisulfid nicht in eine Kategorie für Kanzerogene eingestuft.

**Keimzellmutagene Wirkung. **Es liegen keine Hinweise auf eine genotoxische Wirkung und kein entsprechender Strukturverdacht vor. Daher wird Allylpropyldisulfid nicht in eine Kategorie für Keimzellmutagene eingestuft.

**Hautresorption. **Es gibt keine Studien zur dermalen Aufnahme, keinen systemischen NOAEL und keine Daten zur Löslichkeit. Daher kann die Hautresorption nicht bewertet werden und der Stoff wird weiterhin nicht mit „H“ markiert.

**Sensibilisierende Wirkung. **Nur in einer Publikation in der über positive Epikutantestreaktionen auf Allylpropyldisulfid beim Menschen berichtet wird, werden diese Ergebnisse durch tierexperimentelle Untersuchungen gestützt. Aufgrund der chemischen Strukturanalogie zu Diallyldisulfid kann eine sensibilisierende Wirkung von Allylpropyldisulfid angenommen werden. Jedoch gibt es trotz ausgeprägter Exposition in lebensmittelverarbeitenden Betrieben keine Evidenz für eine sensibilisierende Wirkung. Daher wird Allylpropyldisulfid weiterhin nicht mit „Sh“ markiert. Zur atemwegssensibilisierenden Wirkung von Allylpropyldisulfid liegen keine Untersuchungen vor. Es erfolgt daher weiterhin keine Markierung mit „Sa“.
